# Editorial: Meat packaging and preservation: application of green nanoparticles and other bio-preservatives

**DOI:** 10.3389/fnut.2024.1507181

**Published:** 2024-11-07

**Authors:** S. M. E. Rahman, Jun Wang, Huhu Wang

**Affiliations:** ^1^Department of Animal Science, Bangladesh Agricultural University, Mymensingh, Bangladesh; ^2^College of Food Science and Technology, Guangdong Ocean University, Zhanjiang, China; ^3^College of Food Science and Technology, Nanjing Agricultural University, Nanjing, China

**Keywords:** meat, processing, green nanoparticles, bio-preservatives, quality, safety

Growing populations, urbanization, and changing diets have led to a steady global need for meat products. The meat sector must respond to this rise to maintain a healthy and sustainable food supply. Maintaining meat quality and safety throughout the supply chain is a major challenge in preservation. Traditional methods like freezing and refrigeration have limitations, including energy consumption and risk of bacterial contamination. In recent years, there has been increased interest in new methods for meat packaging and preservation, especially using green nanoparticles and bio-preservatives. Natural green nanoparticles show tremendous potential for preserving meat. These tiny particles have antibacterial, antioxidant, and barrier properties. Silver nanoparticles can prevent the proliferation of foodborne bacteria, hence extends the shelf life of meat products (1). Chitosan, a natural polymer from chitin, is used as a coating to improve packaging sheets, preventing moisture loss, and microbial contamination (2). Using green nanoparticles and bio-preservatives meets the growing consumer demand for eco-friendly food products. These materials are generally safe for humans and cause less environmental harm than traditional synthetic preservatives.

At the outset of this Research Topic, we had written an overview where we were looking original research, review, short communication, case report, and any perspective articles relating to the application of green nanoparticles and bio-preservatives in topics such as:

- Meat preservation & packaging
- Meat quality & shelf life
- Meat safety
- Meat margination
- Value addition to meat and meat products
- Functional meat products
- Phytochemicals in meat and meat products.

So far four articles are published in the final Research Topic highlighting Meat preservation, Value addition to meat and meat products, Meat safety and shelf life but did not cover the area of utilizing green nanoparticles.

Bao et al. wrote on article on “*Effect of freezing-thawing on the quality changes of large yellow croaker treated by low-salt soaking during frozen storage*,” where they looked into how low-salt soaking impacts the quality of large yellow croaker (LYC) while it's stored in the freezer. The results showed that using a low-salt treatment greatly enhanced the quality of LYC when compared to not using any salt at all. Salted LYC showed improved yield, enhanced water retention, and preserved a better texture. The study revealed that using a low-salt treatment helped to slow down the decline in muscle quality during the freezing and thawing process. Although adding salt accelerated certain biochemical processes, all things considered, this approach offered a viable way to maintain the quality of LYC during frozen storage. These results provide key information for improving the transportation, processing, and storage of aquatic products.

Zhao et al. demonstrated how different sterilization methods affect the shelf life and properties of fermented pork jerky. The researchers examined six sterilization methods: boiling, pasteurization, medium-temperature steam, high-temperature steam, ultrasonic, and UV sterilization. Medium-temperature steam sterilization was the best way to lower microbial counts while keeping flavor and texture balanced, outperforming other methods. Since this procedure was highly effective in eliminating S. aureus, a common foodborne pathogen, the product was determined to be safe. While various sterilization methods also helped to improve shelf life, medium-temperature steam sterilization performed better in terms of overall quality. In addition, the method effectively controlled two key markers of food deterioration: total volatile basic nitrogen (TVB-N) and Thiobarbituric acid reactive substances (TBARS). All things considered, medium-temperature steam sterilization is an easy way to preserve fermented pork jerky, providing a safe and tasty snack for customers.

In the review article “*Properties and physiological effects of dietary fiber-enriched meat products: a review*” Mishra et al. comprehensively explores the potential benefits of incorporating dietary fiber into meat products. Dietary fiber, a non-digestible carbohydrate component obtained from plants, has numerous health benefits, including a lower risk of chronic diseases like cardiovascular disease, diabetes, and certain cancers. Researchers and food manufacturers hope to create more nutritious and useful meat products by integrating dietary fiber, which will meet the growing demand for healthier food options. The review dives into numerous areas of dietary fiber, such as its sources, physiological effects, and use in meat processing. It emphasizes the beneficial effects of dietary fiber on the physicochemical qualities, chemical composition, and sensory characteristics of meat products. The review also explores the potential of dietary fiber-enriched meat products to contribute to better human health and tackle the growing need for a healthier diet.

In another study Zhang et al. investigated the effects of plasma-activated water (PAW) on Vibrio parahaemolyticus, a harmful seafood pathogen. This study focused on how PAW works in contaminated shrimp and pure cultures. The researchers found that PAW significantly reduced the vitality of V. parahaemolyticus. PAW treatment damaged the bacterial cell membrane, causing leakage of intracellular components and cell death. Bacterial cells produce reactive oxygen species (ROS), disrupting their antioxidant defense and causing oxidative stress. Furthermore, the analysis showed that PAW treatment changed the expression of genes related to cell membrane integrity and biofilm production. Thus, PAW has the ability to significantly reduce shrimp contamination with V. parahaemolyticus. This suggests that PAW might be an effective method to fight this virus in the seafood industry.

While our Research Topic concentrated on the application of green nanoparticles across various facets of the food chain, we maintained a degree of flexibility. Because our topic span was intended to be broad. We encourage the submission of papers relevant to value addition, preservation, meat quality and safety, shelf life, etc. We believe the four submitted papers effectively fulfilled our objectives in this regard. But there is potential for future collaboration on green nanoparticles in wet meat due to its increasing popularity.
